# Analysis of the Cross-Linking Reaction of Lignin with Triethyl Phosphate by MALDI-TOF and ^13^C NMR

**DOI:** 10.3390/polym9060206

**Published:** 2017-06-04

**Authors:** María Cecilia Basso, Antonio Pizzi, Luc Delmotte, Soliman Abdalla

**Affiliations:** 1 LERMAB, University of Lorraine, ENSTIB-27 rue Philippe Seguin, 88051 EPINAL Cedex 9, 54000 Nancy, France; cecilia-c-c@hotmail.com; 2 Department of Physics, King Abdulaziz University, Jeddah, P.O. Box 80203, 21589 Jeddah, Saudi Arabia; smabdullah@kau.edu.sa; 3 IS2M, Institut de Science des Matériaux de Mulhouse, CNRS LRC 7228; 15, rue Jean Starcky, BP 2488, 68057 Mulhouse, France; luc.delmotte@uha.fr

**Keywords:** lignin, triethyl phosphate, glycerol, catechol, ^13^C NMR, MALDI-TOF, polymerization and cross-linking

## Abstract

The reaction of condensation and cross-linking of desulfurized kraft lignin with triethyl phosphate (TEP) was explored. Catechol, a simple model of the aromatic ring of lignin, and glycerol, a model compound of the aliphatic hydroyl groups of the side chain of lignin, were employed under similar reaction conditions. Solid state cross-polarisation/magic-angle spinning (CP-MAS) ^13^C NMR and matrix assisted laser desorption ionization time-of-flight (MALDI-TOF) spectroscopy studies showed that polycondensation occurs on phenolic hydroxyl groups of lignin, as well as on aliphatic hydroxyls groups of its side chain. The reactions appear to be favoured by higher temperatures and in the presence of ammonia. Preliminary adhesion tests on wood shown good hydrophobicity properties of the surface treated with lignin-TEP-based resin. Initial application tests carried out at high temperature demonstrated as good performance as metallic coating.

## 1. Introduction

Nowadays, the utilisation of renewable resources in industry is essential for sustainable development. Thus, a considerable amount of research focuses on the preparation of thermosetting polymers based on natural products to partially, or even totally, replace those derived from petrochemical feedstocks [1,2,3,4]. 

Lignin is a phenolic, three-dimensional, cross-linked polymer composed of phenylpropanoid units linked to each other by C–O–C and C–C bonds. It represents the second most abundant natural polymer on Earth, being found in plant tissues where its role is in cementing cellulose. Lignin constitutes a waste material from the pulp and paper industry, and it is mainly used (~95%) as fuel for the energy balance of pulping processes [5,6,7,8]. Alternative industrial applications of lignin (~5%) include its use as dispersant, as a binder for wood and particleboard, or as an additive, e.g., for cement or for asphalt, as well as its utilization for animal feed and for pellets used for fuel, etc. Moreover, the production of vanillin by oxidation, mostly used as flavouring or as building blocks in the pharmaceutical industry, represents other current utilizations of lignin [9,10].

However, thanks to its abundance and high functionality, lignin presents diverse potential high-value applications which have encouraged numerous scientific researches focusing on its chemical modification [11,12,13,14,15,16,17,18,19]. For example, hydroxymethylation of lignin by reaction with formaldehyde or even with a non-toxic aldehyde like glyoxal, leads to its use in wood adhesives. Furthermore, the hydroxyl groups of lignin can be functionalized, among others methods, by oxypropylation, yielding new polyols which could be used in the production of biobased PU or epoxy foams, by esterification to synthesize polyester, epoxy resins, and elastomeric materials, or by reaction with isocyanate, leading to obtaining new biobased polyurethane materials. Avérous et al. [20] present a good review of the more significant and promising works in this field.

Recently, Basso et al. [21] have proven a new and interesting reaction of oligomerization and cross-linking of triethyl phosphate (TEP) with condensed (flavonoid) tannins which yields highly thermo-stable materials.

In this context, the aim of this research is to obtain novel thermosetting resins from lignin and TEP, which are able to produce new bio-based and heat-resistant paints, lacquers, coatings, resins, and adhesives [22]. The potential of lignin to polymerize by reacting with TEP is extensively evaluated by solid-state cross-polarization/magic-angle spinning (CP-MAS) ^13^C NMR and matrix assisted laser desorption ionization time-of-flight (MALDI-TOF) analysis. As an application example, initial tests were carried-out on beechwood specimens by measuring by the sessile water drop method the compactness and adhesion of the polymer surface so formed. Furthermore, preliminary coating tests on metallic surface were carried out at high temperature. The findings are exposed in this work.

## 2. Materials and Methods

### 2.1. Preparation of Resins

Catechol (purity > 99%) and glycerol (purity > 99.5%) were used as simple model compounds of lignin. They were supplied by Sigma Aldrich (Saint Louis, MI, USA).

The basic unit of lignin is a phenylpropane. Thus, it possesses an aliphatic chain and a phenol-like aromatic ring. To isolate the possible reactions that can occur on the aliphatic chain and on the aromatic phenol ring of lignin it was decided to use two separate model compounds [23]: glycerol, for the aliphatic chain, and guaiacol and catechol for the aromatic part. As the aliphatic part of lignin contains one hydroxyl group, or can even present more than one hydroxyl group, glycerol is an acceptable model for this part. As guaiacol cannot not polymerize with TEP, as it contains one hydroxyl group only, catechol (ortho-diphenol) was used as a model of the aromatic part of lignin. 

The commercial lignin used was a desulfurized softwood kraft lignin, namely Biochoice kraft lignin supplied by Domtar Inc. (Montreal, QC, Canada) from their Plymouth, North Carolina mill (Plymouth, NC, USA).

Ammonium hydroxide (25%) and triehtyl phosphate were purchased at Acros Organics (Geel, Belgium).

Several experiments were carried out according to the mixture of components shown in Table 1.

All of the samples presented in Table 1 were prepared by mixing the reactives at room temperature. When NH_3_ was used, it was added at the end. No additional catalysts were used. Then, these blends were put into a ventilated oven preheated at 90, 180, or 220 °C, according to Table 1, overnight. Samples were prepared and analysed in triplicate.

### 2.2. Characterization of Samples

#### 2.2.1. CP-MAS ^13^C NMR Analysis

The samples S0, S1, and S2 were ground finely for nuclear magnetic resonance (NMR) analysis. Solid-state CP-MAS (cross-polarization/magic angle spinning) ^13^C NMR spectra of the fine powders obtained were recorded on a Bruker AVANCE II 400 MHz spectrometer (Canteleu, France) at a sample spin of 12 kHz. Chemical shifts were calculated relative to tetramethyl silane (TMS). The rotor was spun at 4 kHz on a double-bearing 7 mm Bruker probe. The spectra were acquired with 5 s recycle delays, a 90° pulse of 5 µs, and a contact time of 1 ms. The number of transients was 3000.

#### 2.2.2. MALDI-TOF Analysis

The samples for matrix assisted laser desorption ionization time-of-flight (MALDI-TOF) analysis were prepared by first dissolving 5 mg of the resins in 1 mL of acetone. Then 10 mg of this solution is added to 10 µL of a 2,5-dihydroxy benzoic acid (DHB) matrix. The locations dedicated to the samples on the analysis plaque were first covered with 2 µL of a NaCl solution 0.1 M in 2:1 *v*/*v* methanol/water, as an analysis enhancer, and pre-dried. Then 1 µL of the sample solution was placed on its dedicated location and the plaque dried again. The reference substance used for the equipment calibration, up to 2000 Da, was red phosphorus.

MALDI-TOF spectra were obtained using an Axima-Performance mass spectrometer from Shimadzu Biotech (Kratos Analytical Shimadzu Europe Ltd., Manchester, UK) using a linear polarity-positive tuning mode. The measurements were carried out making 1000 profiles per sample with two shots accumulated per profile. The spectra precision is of ± 1D.

#### 2.2.3. Wood Surface Coating Application

A resin prepared by mixing 60 g of lignin, 100 g of TEP, and 1 g of glycerol was evaluated for a coating application on the surface of beechwood pieces. First, the resin was spread over each surface with a spatula, around 1.5–2.5 kg/m^2^. Then, the surface was covered with a silicone sheet and the piece was placed on an oven at 180 °C, with a weight of about 1.5 kg on top. 

The hydrophobicity of the surfaces was examined by measuring the contact angle of water on the treated surface by the sessile water drop method. Images were acquired with an Easy Drop contact angle apparatus, using Drop Shape Analysis software (KRÜSS GmbH, Hamburg, Germany). Contact angles during 10 min after the water drop laying were recorded. Untreated beechwood pieces were used as a control.

#### 2.2.4. Metallic Surface Coating Application

A resin was prepared by mixing 2.5 g of lignin, 6 g of NH_3_ (25%), 9 g of water, 2.5 g of TEP, and 0.15 g of glycerol at room temperature. This resin was evaluated for coating application on aluminum plates. First, the resin was applied on the metallic surface and the material was dried at 80 °C. After cooling, the plate was exposed to a temperature of 435 (± 5) °C for 30 min. A cross-cut test, based on the NF EN ISO 2409 standard, was carried out after washing with hot water. For this, the coating was cut through to the metallic substrate with a razor in order to produce edges from which the coating may then be lifted. The cutting pattern consisted of a 10 × 10 grid of 1 mm × 1mm. A strong adhesive tape was applied over the cut area and tightly pressed. Then the tape was then rapidly pulled up. This operation was repeated three times. Finally, the coating was examined, after washing with hot water again, in order to determine the number of blocks removed, thus constituting a method of evaluating adhesion.

## 3. Results

Samples produced from the reaction of lignin and TEP at 180 and 220 °C (S1, S2, S8, and S9) yielded rigid, hard, dark solids which are practically insoluble in acetone. Similar products were obtained from glycerol and TEP at 180 °C (sample S5) and from the reaction of catechol (*o*-diphenol) with TEP at the same temperature (samples S3 and S4). Finally, a flexible black semi-solid was observed when the sample including lignin and TEP (S6) was treated at 90 °C, which is insoluble in water, but soluble in acetone, while the aspect of sample S7, formed by reacting glycerol and TEP, was almost the same before and after thermal treatment at 90 °C, namely, a transparent viscous liquid.

### 3.1. CP-MAS ^13^C NMR Analysis

**Figure 1:**
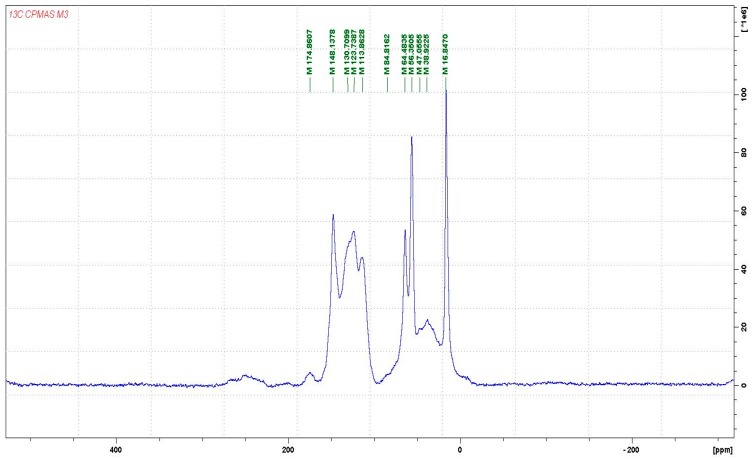
CP MAS ^13^C NMR spectrum of the products obtained by the reaction lignin + TEP + NH_3_ at 180 °C (sample S2).

**Figure 2:**
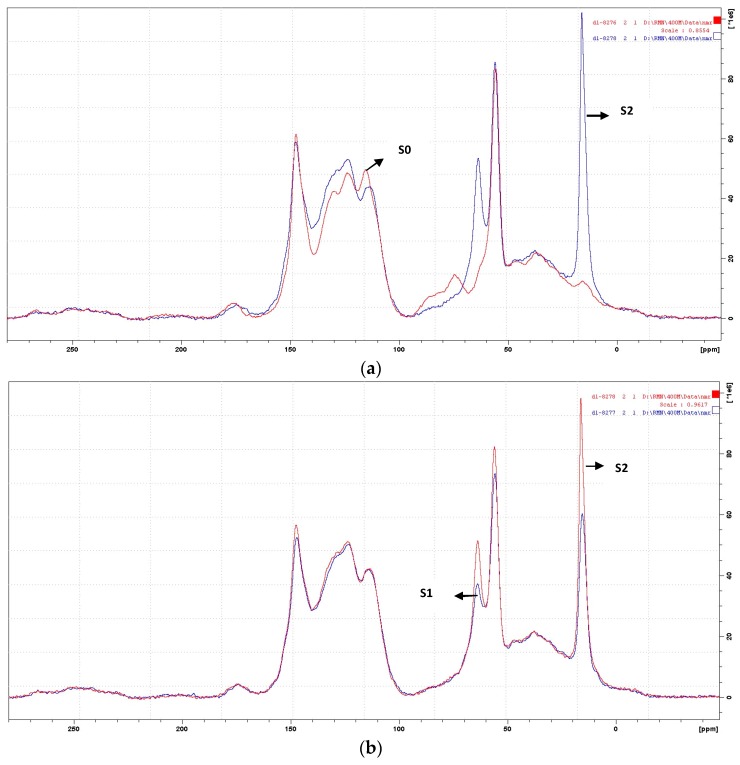
(**a**) The superposition of the CP MAS ^13^C NMR spectra of the original untreated lignin (sample S0) and the products obtained by the reaction lignin + TEP + NH_3_ at 180 °C (sample S2); and (**b**) the superposition of the CP MAS ^13^C NMR spectra of the products obtained by the reaction of lignin + TEP + NH_3_ (sample S2) and lignin + TEP (sample S1), both at 180 °C.

The CP MAS ^13^C NMR spectrum of the products obtained by the reaction lignin + TEP + NH_3_ at 180 °C (sample S2) is presented in Figure 1. The superposition of the CP-MAS ^13^C NMR spectra of samples S2 and S0 (original untreated lignin) and the superposition of the spectra of samples S2 and S1 (lignin + TEP, 180 °C) are shown in Figure 2a,b, respectively.

**Scheme 1:**
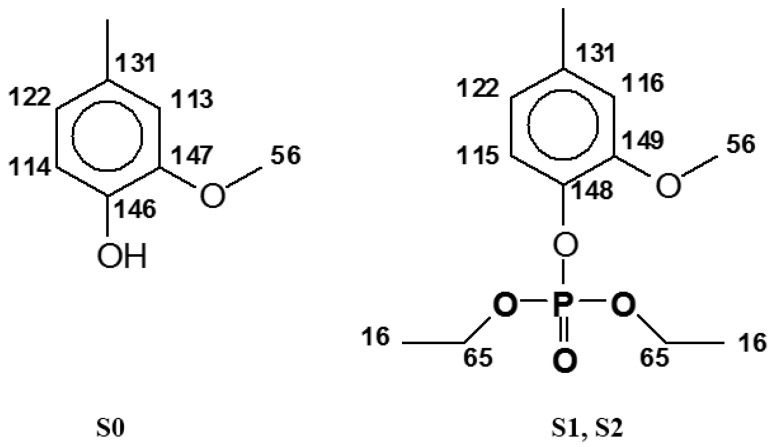
Repeating units and fractions.

The shifts in the ppm characteristic of S0, S1, and S2 can be interpreted according to the two following formulas (Scheme 1):

Looking at the spectra of samples S0 and S2, several differences can be noticed. The decrease of the peak at 147 ppm occurs mainly due to the reaction of TEP on the phenolic –OH groups of some lignin units, the indication being that one of the reactions take place on this site.

Conversely, the peak at 75 pm corresponds of the aliphatic –CH_2_OH of the side chain of the lignin unit. This band disappears in S1 and S2 spectra, indicating that TEP has also reacted on the aliphatic –OHs as well. 

The considerable increases at 65 and 16 ppm for the samples S1 and S2 corresponds to the TEP reaction; theses shifts remain the same to no matter which lignin site TEP is linked to. 

Moreover, the shifts observed at 147 ppm and even more at 56 ppm can be assigned to some demethylation of the lignin during reaction. The peak at 115 ppm decreases due to an internal rearrangement of the lignin leading to β–5 and 5–5 coupling between units of lignin. This causes the peak at 115 ppm to decrease and the one at 128 ppm to increase slightly (with a shoulder at 127–128 ppm). 

The shifts at 65 and 16 ppm of TEP increase considerably in the S1 and S2 cases, and remain the same no matter which lignin site TEP is linked to. The peak at 174 ppm is that of an organic acid or ester: it is a trace, most probably an impurity.

Finally, the peaks at 27–30, 37–38, and 47 ppm do not change. They belong to the original lignin. Of these the peaks at 27–30 and 37–38 ppm are assigned to the carbon of the aliphatic chain of a lignin unit [24,25,26]. Thus, the peak at 27–30 ppm belongs to a CH_3_ or to a –CH_2_– of this chain and the 37 ppm peak to a CH_3_ group attached to a conjugated double bond [25]. The peaks that do not change in the range 14.4–29.6 ppm have been assigned to the CH_2_ and CH groups of the lignin aliphatic lateral chain [24].

**Figure 3:**
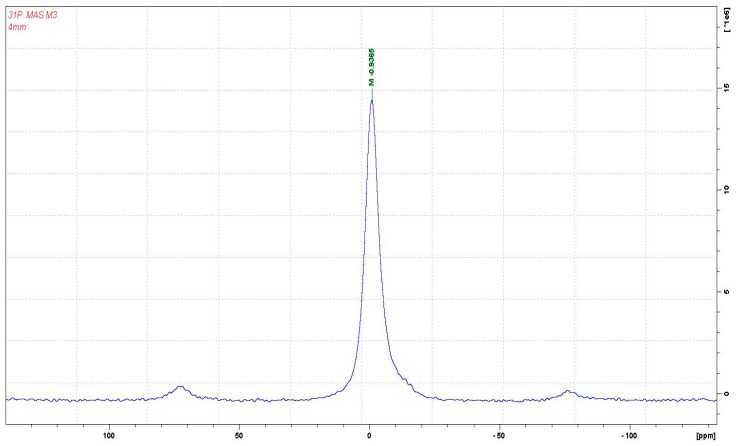
^31^P spectrum of the products obtained by the reaction lignin + TEP + NH_3_ at 180 °C (sample S2).

According to Figure 2b the reaction appears to be favored with ammonia present. Figure 3 displays the ^31^P spectrum of the sample obtained by the reaction of lignin + TEP + NH_3_ at 180 °C (sample S2). The peak at 0 ppm corresponds to the orthophosphate ion (in particular, at −1 ppm occurs the unreacted TEP). According to the shift tables for P NMR [27], the signal observed between −12 and −15 ppm and also at 68 ppm can be attributed to structures of the type O=P(R)(OR)_2_. As a consequence one can recognize the existence of O=P(R)(OR)_2_, O=P(OR)_2_OEt, or O=P(OR)_2_OMe species where R is an aromatic or alkyl group.

The peak at −72 ppm (a wide band between −69 and −75 ppm) can be assigned to a structure of type P(OR)_5_. However (EtO)_5_P should give a signal at −71 ppm, thus, the peak observed is probably due to this latter one. This structure could also explain the peak at −12/−15 ppm (because the signal range for this structure can occur between −5 and −75 ppm). Equally, one could postulate the existence of structures of the O=P(OR)_3_ type, which should occur in the range 0 to −20 ppm. However, in such a case R could be either Et or lignin. Structures of the type (PhO)_3_PO show, in general, a signal at −18 ppm while the peak observed in the spectrum here is at −12/−15 ppm, this being partially hidden by the larger peak. However, it could be shifted due to the particular chemical environment. 

### 3.2. MALDI-TOF Analysis

The MALDI-TOF spectra of the original untreated lignin and their interpretation are presented as Supplementary Materials (Figures S1–S3 and Table S1). 

On the one hand, the spectrum of sample S4, which was obtained from the reaction of catechol + TEP + NH_3_ at 180 °C, in its interpretation are shown in Figure S4 and Table S2 (attached as Supplementary Materials to this article), respectively. These results demonstrated that TEP reacts with catechol, which was used as a simple model compound of the aromatic ring of lignin. 

On the other hand, glycerol was employed as a model of the aliphatic hydroxyl groups of the side chain of lignin. The spectra of the sample S5, which was prepared from of glycerol + TEP at 180 °C, are presented in Figures S5–S7. Some oligomer species formed are listed in Table S3. According to these results, the reaction of TEP occurs on aliphatic –OH groups as well. 

**Figure 4:**
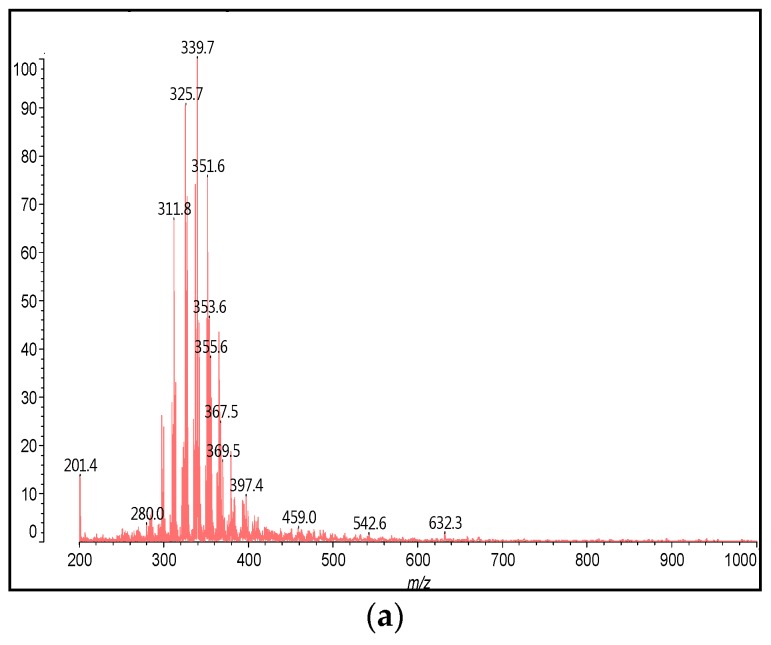

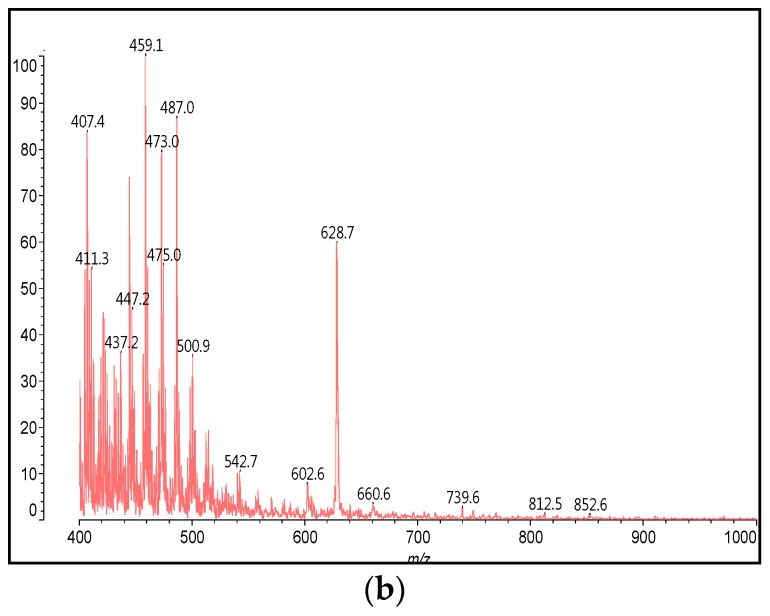
MALDI-TOF spectra of the lignin+TEP reaction at 220 °C (sample S8): (**a**) with an ion gate at 200 Da; and (**b**) with an ion gate at 400 Da.

The MALDI-TOF spectra of products obtained from de reaction lignin + TEP at and 220 °C is shown in Figure 4a,b. The spectra for the same reaction at lower temperature, i.e., 90 and 180 °C, are presented in Figures S8 and S9, respectively (attached as Supplementary Materials). The products of different reactions described previously in Section 3.1 were identified in these spectra.

**Scheme 2:**
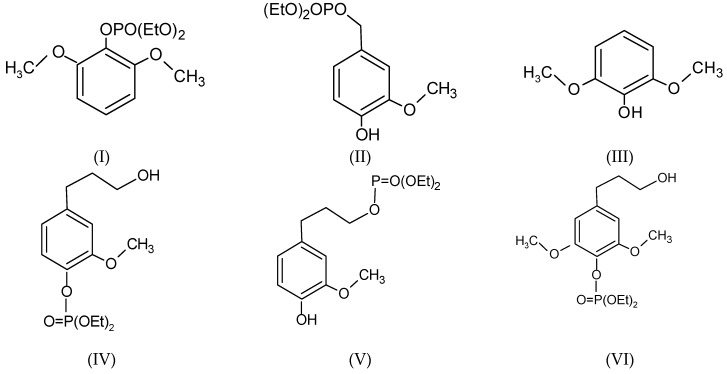

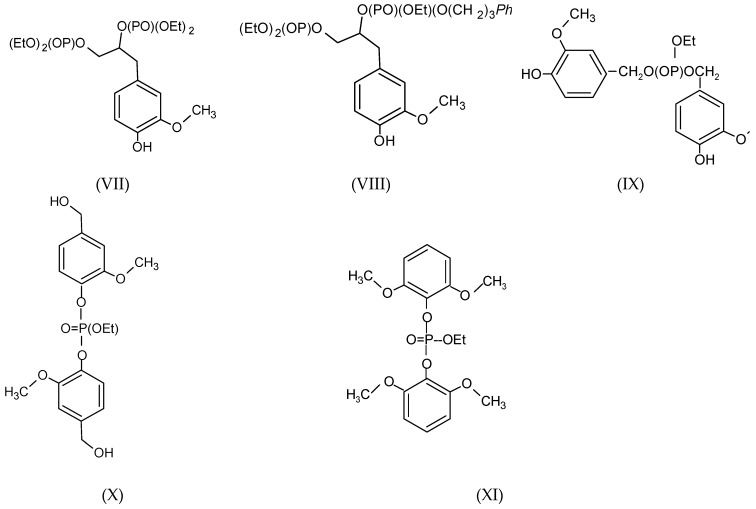
Proposed structures of some oligomers detected by MALDI-TOF for the reaction of lignin+TEP.

TEP is able to react on the aromatic hydroxyl groups of lignin, but also on the aliphatic hydroxyl groups of its side chain. Thus, the peaks found at 312–313 (with Na^+^) and 290 Da (without Na^+^), which have been observed no matter what temperature is applied (90, 180, or 220 °C), are assigned to structures I and II (Scheme 2). The first one comes from the reaction of TEP with syringyl ring where the aliphatic chain side has been split (structure III). 

The peak at 340 Da can be attributed to structures IV and/or V, where TEP reacted with the aromatic or aliphatic hydroxyl groups, respectively. It has been found at 180 and 220 °C, while the peak at 326 Da appears at 220 °C by demethylation of these species.

Other probable structures are VI, at 370 Da (356 Da by demethylation), VII, at 492 Da and 628 Da, by reaction of TEP on the aromatic OH of this last structure. Another possibility for the peak at 628–629 Da is structure VIII.

The peaks at 421, 407 (by demethylation of the first one) and 398 Da (without Na^+^) have been detected at 90, 180, and 220 °C. They are assigned to the structures coming from the reaction of TEP with two monomers of lignin (structures IX, X, XI). Again, the reaction can take place on the aliphatic or aromatic OHs.

The structures included herein are just some possible structures proposed. Alternative isomers can also exist.

**Figure 5:**
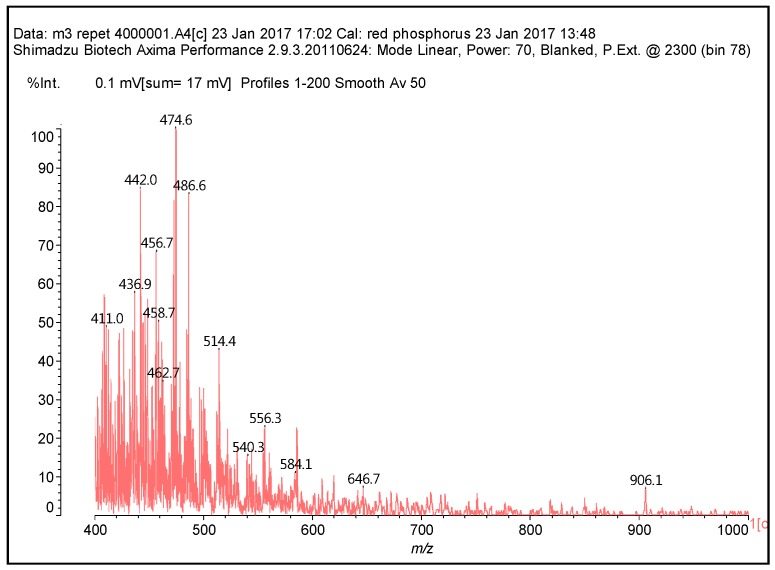
MALDI-TOF spectrum of the lignin + TEP + NH_3_ reaction at 180 °C (sample S2), with an ion gate at 400 Da.

**Figure 6:**
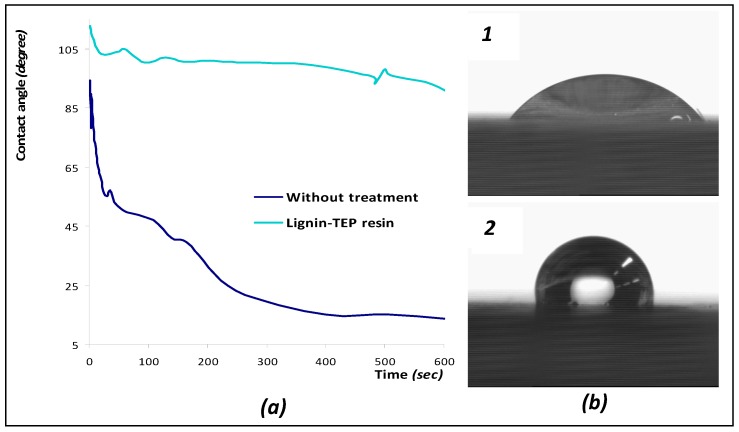
(**a**) The water dynamic contact angle of the lignin-TEP-based resin-coated surface and of the untreated beech sample used as a reference; and (**b**) the water drop shape after 60 s on: (1) untreated beech (reference) and (2) the lignin-TEP-based resin-coated surface.

The reaction of TEP with three or more units of lignin is observed when the higher reaction temperatures are used (180 and 220 °C, Figure 5 and Figure 6). For example, the peak at 526–529 Da (deprotonated or not) corresponds to structures XII and XIII, by reaction of TEP on the aromatic or aliphatic –OHs, respectively, (peaks at 501 and 487 Da by demethylation). 


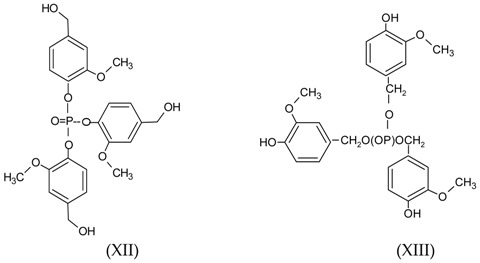


Some other examples of species generated by reaction of TEP on the aromatic and/or aliphatic –OH groups of lignin are shown in Table S4 (presented as Supplementary Materials). Most of them, as well as those described previously, have been observed when the temperature reaction is 180 °C or higher. Consequently, a higher temperature seems to favor the reaction between TEP and lignin. This trend has already been reported for the reaction between TEP and condensed tannins [21].

It is rather evident that cyclic structures exist, generated by intramolecular reactions and corresponding to some of the peaks observed in the MALDI-TOF spectra. Thus, the peak at 311 Da corresponds to a compound of 288 molecular weight (+23 of Na^+^ = 311 Da) in which TEP can form a cyclic structure with two aliphatic hydroxyl groups of lignin units, as follows: 
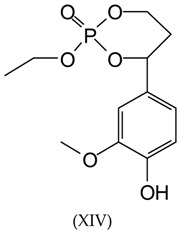


Equally, with rearrangement of some syringyl groups, cyclic structures of the following type occur at 419 Da (structures XV and XVI): 
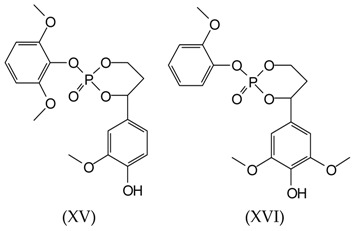


Additionally, a 447 Da peak found for the samples S1, S6, and S8 corresponds to the following structure: 
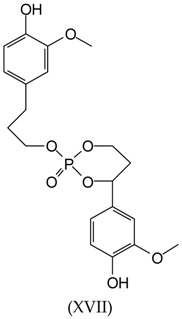


The peak at 463 Da is present in the spectra of samples S1 and S8 and can correspond to several possible structures, cyclic and non-cyclic, composed of two lignin units linked by reaction through a molecule of TEP. The two lignin units can either be linked through the reaction of TEP with the lignin aliphatic –OHs, or through two phenolic hydroxyl groups, or an equal mix of the two. As the molecular weights of structures (XVIII) and (XIX) are of 440, yielding an expected peak of 463 Da these two structures are the two more probable ones as structures (XX) and (XXI), respectively, molecular weights of 438.5 and 438.1 would theoretically yield peaks of 461–462 Da. However, they cannot be excluded as it would be enough that one hydroxyl group is protonated, a likely occurrence, to also yield a peak at 463 Da.


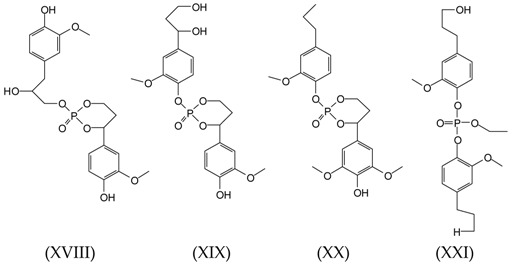


The possibility of a linkage between two lignin units through the TEP reacting with one phenolic and one alcoholic hydroxyl groups of lignin also exist for structure XVII, shown above, represented by the peak at 447 Da. 

Finally, several indications were found regarding demethylation reactions of lignin, which has already been noticed by NMR analysis. For example, the series 501 → 487 → 473 → 459 and 367.5 → 353.6 → 339.7 → 325.7 → 311.8 have been detected in Figure 4a,b.

The MALDI-TOF spectra of products obtained from de reaction lignin + TEP + NH_3_ at 180 °C are shown in Figure 5. The products of different reactions described previously for TEP + lignin have also been observed in these spectra. However, additional species were yielded for the reaction of lignin with TEP and NH_3_. Moreover, the peaks observed for sample S2 prepared at 180 °C, are almost the same as that for sample S9, which was obtained at 220 °C (not shown here).

The possibility that the ammonia used in some of the cases has substituted the phenolic hydroxyl groups of two lignin units also exists. This type of reaction is facile and has already been reported for other phenolic compounds [28].

This would yield structures of the following type: 
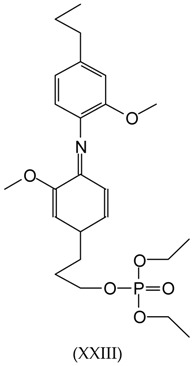


Then, a peak at 489 Da can be found in Figure 5, representing the structure (monoprotonated) of molecular weight 465.5 (465.5 + 23 = 488.5 Da).

Equally, for the peak at 619 Da (without Na^+^): 
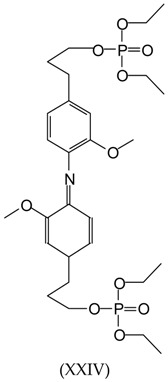


The possibility of the TEP linking to the –NH_2_ group having substituted the phenolic hydroxyl group to form groups of the type Ar–NH(PO)(OEt)_2_ is also present, although this cannot be confirmed with the data available.

### 3.3. Wood Surface Coating Application: Contact Angle Measures with Water

The dynamic contact angle of water on the coated wooden surface and on an untreated sample used as reference, are presented in Figure 6a. Figure 6b shows the shape of water drops after 60 s for the untreated reference and the lignin-TEP-based resin-coated surface.

The contact angles recorded for the surface treated with the lignin-TEP-based resin are much higher than the ones measured for the untreated surface (Figure 6a,b). Moreover, the contact angle values for the untreated surface decrease rapidly during the evaluated period while, for the surface treated with the lignin-TEP resin, they stay almost constant (Figure 6a). According to these results, the polymer formed by the reaction of lignin with TEP reduces the water uptake of the wooden surface, which, with bonding, constitutes one of its possible applications.

### 3.4. Metallic Surface Coating Application: Adhesion Tests

**Figure 7:**
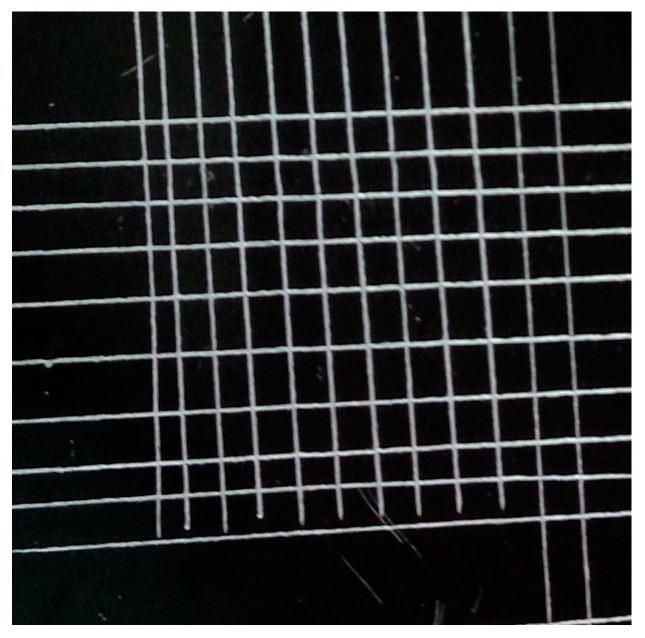
Result of the cross-cut test of the lignin-TEP-based resin-coated aluminum surface.

Figure 7 shows the aspect of the coated surface after the cross-cut test. None of the squares of the lattice are detached and the edges of the lines are smooth. Accordingly, the adhesion was evaluated, pointing out that lignin-TEP-NH_3_-based resins can be applied as metal coatings with high-temperature resistance. 

## 5. Conclusions

A novel reaction of polycondensation and cross-linking of lignin with TEP is described for the first time. The studies showed that TEP reacts on the phenolic hydroxyl groups of lignin but also on the aliphatic hydroxyl groups of its side chain. The reaction appears to be dependent on the temperature. Thus, it is favored certainly from a temperature of 180 °C, yielding insoluble, hardened resins, as well as when ammonia is present, due to additional cross-linking reactions. Good hydrophobicity properties were found for the wood surfaces coated with a lignin-TEP-based resin at 180 °C. Initial application tests carried out at 435 °C on aluminum demonstrated good performance as a metallic coating.

## Figures and Tables

**Table 1:** Compositions and temperature of synthesis of samples S0 to S9, derived from lignin, catechol, glycerol, triethyl phosphate (TEP), and ammonia (NH_3_).

Sample	Temperature (°C)	Composition				
		Lignin 3 g	NH_3_ (25%) 1.5 g	TEP 3 g	Catechol 3 g	Glycerol 3 g
S0	-	X	-	-	-	-
S1	180	X	-	X	-	-
S2	180	X	X	X	-	-
S3	180	-	-	X	X	-
S4	180	-	X	X	X	-
S5	180	-	-	X	-	X
S6	90	X	-	X	-	-
S7	90	-	X	X	-	X
S8	220	X	-	X	-	-
S9	220	X	X	X	-	-

## Supplementary Materials

Figure S1: MALDI-TOF spectrum of original untreated lignin (sample S0), without an ion gate; Figure S2: MALDI-TOF spectrum of original untreated lignin (sample S0), with an ion gate at 200 Da; Figure S3: MALDI-TOF spectrum of original untreated lignin (sample S0), with an ion gate at 400 Da; Figure S4: MALDI-TOF spectrum of the catechol + TEP + NH_3_ reaction at 180 °C (sample S4), without an ion gate; Figure S5. MALDI-TOF spectrum of the glycerol + TEP reaction at 180 °C (sample S5), without an ion gate; Figure S6. MALDI-TOF spectrum of the glycerol + TEP reaction at 180 °C (sample S5), with an ion gate at 100 Da; Figure S7. MALDI-TOF spectrum of the glycerol + TEP reaction at 180 °C (sample S5), with an ion gate at 300 Da; Figure S8. MALDI-TOF spectrum of the lignin + TEP reaction at 90 °C (sample S6): (a) with an ion gate at 200 Da; (b) with an ion gate at 400 Da; Figure S9. MALDI-TOF spectrum of the lignin + TEP reaction at 180 °C (sample S1): (a) with an ion gate at 200 Da; (b) with an ion gate at 400 Da. Table S1: Structures determinate for MALDI-TOF analysis of original untreated lignin, Table S2: Interpretation of peaks of MALDI-TOF spectrum derived from the reaction of catechol + TEP + NH3 at 180 °C. Table S3: Oligomer species formed during the reaction at 180 °C between glycerol and TEP, Table S4: Oligomer species formed during the reaction between TEP and lignin.
