# Supplementary material for: Analysis of the Cross-Linking Reaction of Lignin with Triethyl Phosphate by MALDI-TOF and 13C NMR
Source: Polymers (Basel). 2017 Jun 4;9(6):206. doi: 10.3390/polym9060206 (PMC6431866; doi:10.3390/polym9060206)
Supplement: Supplementary file 1 [file polymers-09-00206-s001.docx]

**Supplementary Materials**:

**Fig. S1**. MALDI-TOF spectrum of the original untreated lignin (sample S0), without ion gate.

**Fig. S2**. MALDI-TOF spectrum of the original untreated lignin (sample S0), with ion gate at 200 Da.

**Fig. S3**. MALDI-TOF spectrum of the original untreated lignin (sample S0), with ion gate at 400 Da.

**Fig S4**. MALDI-TOF spectrum of the catechol+TEP+NH_3_ reaction at 180 ° C (sample S4), without ion gate.

**Fig S5**. MALDI-TOF spectrum of the glycerol+TEP reaction at 180 ° C (sample S5), without ion gate.

**Fig S6**. MALDI-TOF spectrum of the glycerol+TEP reaction at 180 ° C (sample S5), with ion gate at 100 Da.

**Fig S7**. MALDI-TOF spectrum of the glycerol+TEP reaction at 180 ° C (sample S5), with ion gate at 300 Da.

(a)

(b)

**Fig S8**. MALDI-TOF spectrum of the lignin+TEP reaction at 90 ° C (sample S6): (a) with ion gate at 200 Da; (b) with ion gate at 400 Da.

(a)

(b)

**Fig S9**. MALDI-TOF spectrum of the lignin+TEP reaction at 180 °C (sample S1): (a) with ion gate at 200 Da; (b) with ion gate at 400 Da.

**Table S1**. Structures determinate for MALDI-TOF analysis of original untreated lignin.

| **Calculated (Da)** | **Structure** | **Comment** |
| --- | --- | --- |
| 177 |  | With Na^+^ |
| 180 |  | Without Na^+^, deprotonated |
| 198 |  | Without Na^+^ |
| 242/ 265 |  | Without/with Na^+^ |
| 274/ 297 |  | Without/with Na^+^ |
| 304/ 327 |  | Without/with Na^+^ |
| 401 |  | With Na^+^ |
| 420/ 433 |  | Without/with Na^+^ |
| 444 |  | With Na^+^ |
| 443 |  | With Na^+^ |
| 514/ 537 |  | Without/with Na^+^ |
| 541/ 564 |  | Without/with Na^+^ |
| 530/ 552 |  | Without/with Na^+^ |
| 558/ 581 |  | Without/with Na^+^ |

**Table S2.** Interpretation of peaks of MALDI-TOF spectrum derived from the reaction of catechol+TEP+NH_3_ at 180 °C (Ph = phenyl; OEt = -OCH2CH3).

| **Peak(Da)** | **Assignement (with Na^+^)** |
| --- | --- |
| 331 | OH-Ph-OPO(OEt)O-Ph-OH |
| 359 | OH-Ph-OPO(OEt)O-Ph-OEt |
| 405 | (EtO)_2_OPO-Ph-OPO(OEt)_2_ |
| 387 | EtO-Ph-OPO(OEt)O-Ph-OEt |
| 535 | OH-Ph-OPO(OEt)O-Ph-OPO(OEt)O-Ph-OH |
| 873 | OH-Ph-OPO(OEt)O-Ph-OPO(OEt)O-Ph-OPO(OEt)O-Ph-OPO(OEt)_2_ |
| 222 | NH_2_-Ph-NH-Ph-NH_2_ |
| 331 | NH_2_-Ph-OPO(OEt)O-Ph-NH_2_ |
| 359 | NH_2_-Ph-NH-Ph-OPO(OEt)_2_ |
| 359 | OH-Ph-NH-Ph-NHPO(OEt)_2_ |
| 360 | OH-Ph-NH-Ph-OPO(OEt)_2_ |
| 360 | EtO-Ph-OPO(OEt)O-Ph-NH_2_ |
| 387 | EtO-Ph-NH-Ph-NHPO(OEt)_2_ |
| 388 | EtO-Ph-NH-Ph-OPO(OEt)_2_ |

**Table S3.** Oligomer species formed during the reaction at 180° C between glycerol and TEP.

| **Calculated, with Na^+^ (Da)** | **Structure** |
| --- | --- |
| 251 | or  |
| 341 |  |
| 387 | or  or  |
| 268 |  |
| 450 |  |
| 767 |  |
| 906 |  |

**Table S4.** Oligomer species formed during the reaction between TEP and lignin (*Ph*: aryl group; (PO): P=O group)

| **Structure** | **Calculated (Da)**  **(with Na+)** |
| --- | --- |
|    | 477, 449 by demethylation |
|   | 449 |
|    | 356 |
| * **  | 509 (486 without Na) |
| * **  | 661 |
| * **  | 465 |
| * **  | 629 |

* or introduction on the other aliphatic –OH for one or more of the monomers.

** or bond through aliphatic -OHs and aromatic –OHs.
